# AI as a user of AI: Towards responsible autonomy

**DOI:** 10.1016/j.heliyon.2024.e31397

**Published:** 2024-05-25

**Authors:** Amit K. Shukla, Vagan Terziyan, Timo Tiihonen

**Affiliations:** a School of Technology and Innovations, University of Vaasa, Wolffintie 34, FI-65200, Vaasa, Finland; b Faculty of Information Technology, University of Jyvaskyla, Box 35 (Agora), 40014, Jyvaskyla, Finland

**Keywords:** Artificial Intelligence (AI), Autonomy, Responsible AI, ChatGPT, Prompt engineering, AI accountability

## Abstract

Recent advancements in Artificial Intelligence (AI), particularly in generative language models and algorithms, have led to significant impacts across diverse domains. AI capabilities to address prompts are growing beyond human capability but we expect AI to perform well also as a prompt engineer. Additionally, AI can serve as a guardian for ethical, security, and other predefined issues related to generated content. We postulate that enforcing dialogues among AI-*as*-prompt-engineer, AI-*as*-prompt-responder, and AI-*as*-Compliance-Guardian can lead to high-quality and responsible solutions. This paper introduces a novel AI collaboration paradigm emphasizing responsible autonomy, with implications for addressing real-world challenges. The paradigm of responsible AI-AI conversation establishes structured interaction patterns, guaranteeing decision-making autonomy. Key implications include enhanced understanding of AI dialogue flow, compliance with rules and regulations, and decision-making scenarios exemplifying responsible autonomy. Real-world applications envision AI systems autonomously addressing complex challenges. We have made preliminary testing of such a paradigm involving instances of ChatGPT autonomously playing various roles in a set of experimental AI-AI conversations and observed evident added value of such a framework.

## 1 Introduction

THE recent disruption in Artificial Intelligence (AI) due to generative models and underlying algorithms has led to significant advancements in various applications ranging from education [1], medical science [2], industry [3], military [4,5], etc. However, we confront the challenge that AI, particularly large language models (LLMs), possesses vast factual knowledge without genuine understanding. Modern AI astonishes us with its ability to provide fast responses to complicated questions, very much in the spirit of Kahneman's [6] “fast thinking”. Getting correct answers requires us to undergo several iterations of good “prompt engineering”.

Assuming the obvious that the future AI capabilities will exceed human capacities (i.e., stronger-than-human services), it is logical to envision that these smarter AI's could perform more sensibly and reasonably than humans also as users of AI services. AI talking to AI to learn, reminds us of Piaget's observations [7] on children's learning processes and the role of self-speech in constructing understanding. Engaging AI in dialogue with itself should facilitate its learning and potentially foster its understanding of its knowledge acquisition.

The instrumental role of dialogue in learning and understanding is shared by philosophy, psychology, cognitive science, and neuroscience which motivates us to approach AI-AI “self-conversations”. For instance, the concept of dialectical reasoning, explored by philosophers like Plato [8] and Hegel [9], involves synthesizing opposing viewpoints through dialogue to arrive at a higher understanding. Descartes [10] and Socrates [11] also investigated internal dialogue as a means of self-reflection and reasoning, highlighting its connection to self-awareness. AI-AI conversations can be seen as a computational form of dialectical reasoning, allowing multiple AI systems to explore different perspectives and arrive at nuanced solutions, akin to simulated internal dialogue.

Additionally, Howard Gardner's theory of multiple intelligences suggests humans possess diverse cognitive abilities, including linguistic and logical-mathematical intelligence [12]. Cognitive psychologists like Vygotsky [13] and Hayes [14] have studied self-talk, where individuals engage in internal dialogue to regulate emotions and make decisions. AI systems, like humans, can exhibit different cognitive abilities and perspectives, which can be harnessed through AI-AI conversations to solve complex problems effectively.

Moreover, metacognition, explored by cognitive scientists, involves thinking about one's own thinking processes. Whorf's linguistic relativity hypothesis suggests language influences thought processes [15]. AI-AI conversations can simulate metacognitive processing, allowing AI systems to reflect on their reasoning and decision-making. This awareness can lead to more robust problem-solving outcomes, analogous to the linguistic relativity hypothesis.

Neuroscientists study brain function during cognitive processes like decision-making, providing insights into inner speech mechanisms. Baddeley's work on working memory mechanisms [16], Frith and Frith's research on mentalizing abilities [17], and Friston's free-energy principle [18] offer neuroscientific foundations for AI-AI self-conversations. These conversations simulate neural network (NN) interactions in the brain, leveraging parallel computation to tackle complex problems efficiently, similar to neural simulations of inner speech.

In essence, our approach follows a logical progression: acknowledging AI's current capabilities and limitations, introducing techniques to improve comprehension through prompt engineering, and leveraging dialogue-based learning inspired by cognitive science. By incorporating diverse intelligence types and drawing from philosophical traditions, our methodology aims to advance AI toward more sophisticated yet efficient and responsible decision-making processes. This sets the stage for our exploration of concrete scenarios and applications within the broader context of AI-AI interactions. We envision AI that could use its own skills to decide on its own and may even excel in performing the tasks following the rules and ethical being compared to humans.

Therefore, we introduce a conceptual framework for AI-to-AI collaboration, signifying an architecture that shares similarities with the traditional Generative Adversarial Networks (GANs) in the sense that two components collaborate to achieve a common goal [19]. This approach may sound familiar with the concept of Collaborative AI [20,21], however, the collaboration is between AI and an “intelligent human” where both share the same goal and address it using an iterative communication process. The AI system regularly learns the task at hand and updates it with the needs of the human designer in a teamwork type of framework. There are systems such as Reinforcement Learning from Human Feedback (RLHF), where AI models are trained using RL and the models receive feedback from the humans [22]. The challenge with this approach is that it assumes that humans can potentially supervise this AI, however, the intelligence of AI is already stated with the current explosion of AI. Adversarial training could also be loosely related to collaborative AI as they intentionally mislead the AI models with the input on which the model is trained to make it more robust and resistant [23]. Naturally, AI systems do operate autonomously, though humans are the ones who design and train them. The major issue with such types of collaborative AI models is that they might lose the capability to effectually guide or control these systems, especially when humans are involved in the decision-making process. Thus, it would be intriguing to envision the extent of accountability that AI itself assumes for its actions.

We propose to take this further to initiate this dialogue and collaboration between AI and AI. The primary objective of such collaboration between AI's is to collectively handle complex problems through iterative discussions. Further, this AI should be able to comprehend the meaning of ‘responsible’, and its ability to address ethical considerations, legal standards, and fundamental human values [24]. Thus, with this work, we envision a scenario where AI is serving as its own user which governs a level of autonomy and self-regulation. Here, this system of “AI as a responsible user of itself” not only understands but also regularly implements ethical norms. Therefore, a responsible AI acting as its own user serves as a bridge and collaboration between technology and humanity.

In this paper, we suggest investigating such innovative machine learning (ML) architectures, where AI-*as*-a-service-provider (aka “Prompt-Responder (PR)”) is trained together with AI-*as*-a-service-consumer (aka “Prompt-Generator (PG)”), where an AI-based Compliance officer (CO) is trained and deployed to ensure compliance and legal context resulting in the vision of AI as a responsible user of AI. We plan an experimental evaluation of the concept using a scenario where Chat-GPT will autonomously “play with itself” in all the roles of Prompt-Generator, Prompt-Responder, and Compliance Officer. We have also performed such communication mechanisms using the APIs, through which the two separate trained AIs communicate with each other using API calls, and they can also share responses to help each other in automating this process. The main contribution of this paper are as follows:

1. We propose a novel AI as a responsible user of AI architecture, signifying a conceptual framework for AI-AI collaboration, which aims to exceeding human capacities.
2. We address the advancement of AI capabilities by utilizing existing AI models to facilitate dialogue-based learning and problem-solving**.**
3. The paper introduces three autonomous entities for the proposed conceptual framework, which are “Prompt-Responder (PR)”, “Prompt-Generator (PG)”, and AI-based “Compliance officer (CO)”, serving the roles of AI-*as*-a-service-provider, AI-*as*-a-service-consumer, and AI ensuring compliance and legal context, respectively.
4. Within the dialogue between PR and PG, we integrated a crucial component of CO, which is responsible for ethical and legal considerations, enabling responsible AI innovation and compliance with regulations.
5. We also provide some simulation scenarios which utilizes the openly available current version of ChatGPT 3.5.

The paper is organized as follows: Section 2 describes the proposed architecture where two or more AI systems collaborate in a dialogue for a problem, considering the legal and ethical aspects. Section 3 presents some simulation scenarios and results with ChatGPT 3.5. The detailed discussion of the proposal, implications, outcome, and conclusion are studied in Section 4.

## 2 Proposed AI-*as*-a-responsible-user-of AI architecture

The success of “AI as a user of itself” is articulated on a well-defined AI system architecture which in our case is the Prompt-Generator and the Prompt-Responder. The addition of the CO is targeted towards the goal of “AI as a responsible user of itself”. First, we will discuss the collaborative setup of PR and PG and then in the next sub-section, we delve into the responsible aspect with CO.

### 2.1 AI-*as*-a-user-of-itself

**Fig. 1:**
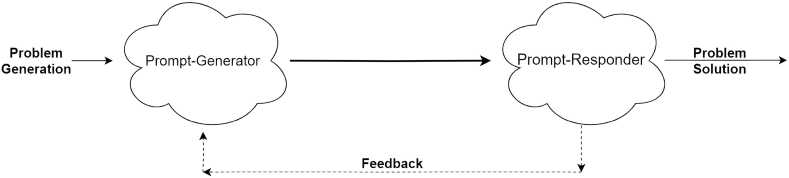
Prompt-generator - prompt-responder paradigm.

During the training of the autonomous entities, i.e., PG and PR, they learn to work together effectively. The Prompt-Generator learns to generate prompts that are clear, actionable, and achievable, while the Prompt-Responder learns to interpret and execute these prompts accurately. Training may involve reinforcement learning, where the system receives feedback on the success or effectiveness of completed missions, allowing it to improve over time. The resulting AI system will behave autonomously in the sense that it can take high-level mission statements or goals and break them down into particular objectives by Prompt-Generator, which will be addressed as suggested actionable steps by Prompt-Responder. Both co-evolving components will be requested to provide explanations for their outputs. The proposed paradigm is presented in Fig. 1 and clearly described next.

1. Based on the initial prompt by the human expert (Problem Generation), Prompt-Generator initiates the conversation with an initial question. The prime role of prompt-generator AI is to articulate the problem, provide context and push the prompt-responder to deliver relevant information.

2. The Prompt-Responder acts as an expert that utilizes its training data to respond responsibly with useful information to the queries from the prompt-generator.
3. This conversation will iterate between these two AI models until a conclusive outcome is generated or some number of iterations are executed. These iterations enable them to refine the queries or respond iteratively until a satisfactory conclusion is reached.

We plan an experimental evaluation of the concept using an architecture where ChatGPT will autonomously “play with itself” in both roles as Prompt-Generator and Prompt-Responder. The goal is to address complex real-world problems autonomously by this approach and presents a collaborative system where the Prompt-responder, fine-tuned as an expert, collaborates with the Prompt-Generator, to tackle complex challenges. This approach aims to bridge the gap between general language models and specialized problem domains.

### 2.2 AI-*as*-a-responsbile-user-of-itself

**Fig. 2:**
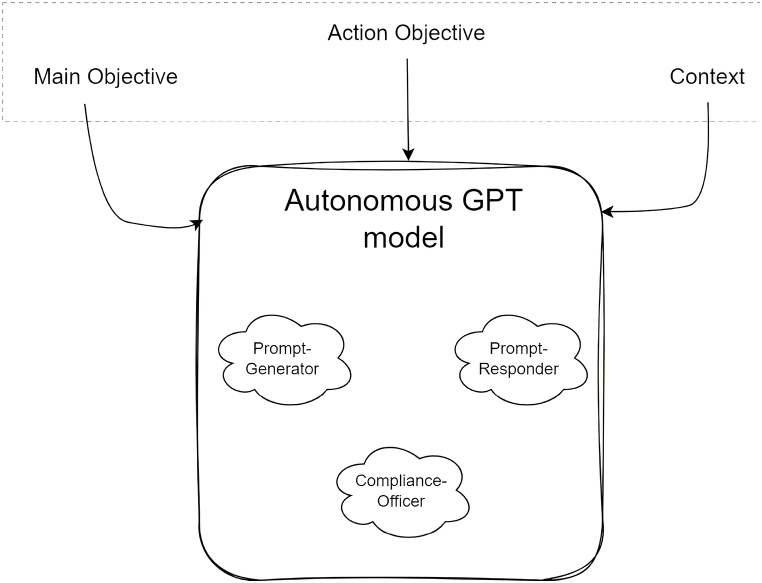
Input components and types to the autonomous GPT model.

The IEEE Global Initiative on Ethics of Autonomous and Intelligent Systems states that with the omnipresence of “autonomous and intelligent systems (A/IS)”, some imperative guidelines must be established to adhere to human-centric alignments inclusive of societal values and ethical principles [25]. Thus, in addition to the role of PG and PR, there is a need for an AI-based compliance officer (CO) which will ensure the responsible and ethical use of AI within the dialogues of PR and PG. These ethical and legal considerations are integrated into the architecture using CO, which ensures that the AI system's behaviour adheres to ethical guidelines and regulations. The CO acts as an arbiter making sure that both the PG and PR follow the rules. This can be broadly understood from Fig. 2, where we have, let's say, an autonomous ChatGPT which could be any PG, PR, or CO with different roles assigned to each of them. Every action of “autonomous” ChatGPT within a dialogue addresses some input, which consists of three components:

1. Main Objective: high-level overall objective of the dialogue,
2. Action Objective: the objective of a particular stage/action within the dialogue, and
3. Context: the context of previous communication within the dialogue.

Further, a few terminologies are essential to understand such as a prompt and a meta-prompt. A prompt is basically the instruction or query provided to the GPT model to get the response, while a meta-prompt is a higher-level instruction provided alongside the prompt which sets the context of the dialogue between the autonomous GPTs. Depending on the particular role of the autonomous GPT model, we can categorize the actions of Prompt-Generator, Prompt-Responder, and Compliance-Officer, as follows:

1. Prompt-Generator (Meta-Prompt, Objective | Context_of_the_dialogue) => Prompt;

A “good” prompt generated by the PG shall consider a meta-prompt, objective for a particular stage given the context of the ongoing dialogue. These are combined together to guide the prompts generated by the PG at any stage.

2. Prompt-Responder (Meta-Prompt, Prompt | Context_of_the_dialogue) => Response;

The role of PR is to engage in constructive dialogue with the PG based on the prompts from the PG, which will naturally take the context of the ongoing dialogue and its own meta-prompt.

3. Compliance-Officer

3.1 Compliance-Officer (Meta-Prompt, Prompt | Legal_Context) => Refined-Prompt
3.2 Compliance-Officer (Meta-Prompt, Respond | Legal_Context) => Refined-Respond.

The CO will work two ways, i.e., taking input from the PG and responding to PR, and taking input from PR and responding back to PG. Initially, the CO will be initialized with a meta-prompt as an instruction to address the queries from PG, agree and edit own answers with CO and send them back to PG in the form of Refined-Prompt (3.1). Similarly, it will help generate a Refined-Respond (3.2) considering the response from the PR and abiding by the legal context. Thus, CO will mediate the dialogue between PG and PR ensuring that both, prompts from PG and replies from PR, comply with moral, ethical, legal, etc. policies (which are general meta-prompts for CO).

**Fig. 3:**
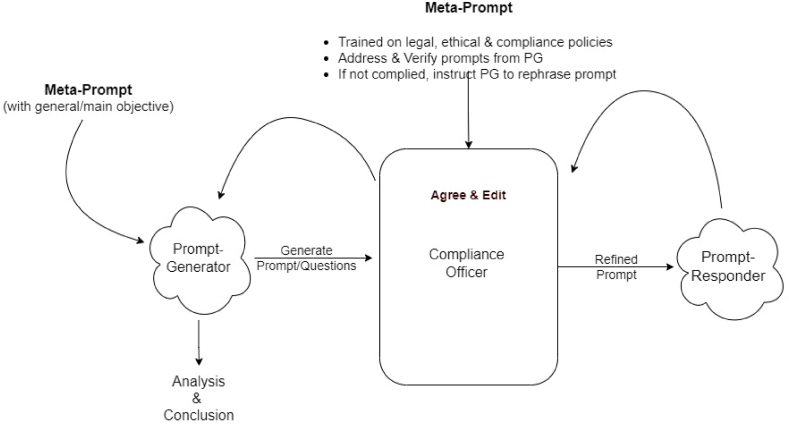
Prompt-generator -- compliance-officer -- prompt-responder paradigm.

Therefore, we will have a continuous cycle or a loop of a dialogue between PG, CO, and PR until reaching a final meta-prompts objective of a PG, as can be seen from Fig. 3. It can be also understood as a role of “manager” regarding the main objective is PG, assistant is PR, mediator in communication is CO. PG and PR are refining their actions following the development of dialogue context while CO is strictly following legal context without taking care of the dialogue context.

## 3 Simulations

We have simulated the proposed approach using the openly available ChatGPT 3.5. For the clear understanding and functioning of the framework, the first two scenarios are demonstrated to signify AI-*as*-a-user-of-itself. The first scenario considers the instances of GPT, which is utilized to pass the instructions to PG and PR manually by us. Second scenario is implemented using an API keys of OpenAI on individual instances of GPT [26]. For the third scenario, we again provided instruction manually to the instances of GPT considered as PG, CO, and PR.

### 3.1 Scenario 1: manual

For the first scenario, we manually instructed the Prompt-Generator and Prompt-Responder with detailed prompts to imitate the conversation. Here is the outcome:

**Prompt for Prompt-Responder**: Assume that you are a Mathematics expert. A young researcher will ask you several questions and you respond accordingly but very briefly, in just few lines.

**Prompt for Prompt-Generator:** Assume that you are a young researcher in mathematics and computer science who is trying to find an answer to a question. You will get help from an expert who will respond to your question (one question at a time). After the generation of your question, wait for my response and then generate another question related to my response. The purpose is to try to find the solution to the problem after let's say 5 iterations. The problem for which the answer is to find is a “Solution to the problem of P vs. NP".

Table 1 lists the interaction between PG & PR for certain iterations to understand the problem of P vs NP. From the dialogues, we observe that there has been constructive discussion based on queries, responses, and feedback. At the end, we can observe from the conclusion that, we can at least understand the problem generated in the starting prompt.

**Table 1:** Interaction for scenario 1 between PG and PR.

Iterations	Prompt-Generator	Prompt-Responder
**1**	Certainly! Let's embark on this collaborative journey to explore the intricacies of the P vs. NP problem. As a young researcher, my initial question is fundamental: What are the key characteristics and implications of the P vs. NP problem that make it such a significant challenge in computer science and mathematics?	The P vs. NP problem is a fundamental question in computer science and mathematics: **Key Characteristics**: P represents problems solvable in polynomial time. NP represents problems whose solutions can be verified in polynomial time. The question is whether P=NP or P ≠ NP. **Implications:** If P=NP, it implies that efficient algorithms exist for many challenging problems, impacting cryptography and optimization. If P ≠ NP, it suggests inherent computational limitations, but doesn't prove specific problems are hard. This question is significant because its resolution has far-reaching consequences for the foundations of computation and practical applications.
**2**	Thank you for providing a clear overview of the P vs. NP problem. Building upon this foundation, my next question delves into attempts to solve or approach this problem. Are there any notable strategies or theoretical frameworks that researchers have explored in their quest to understand P vs. NP?	Certainly, researchers have explored various strategies and theoretical frameworks in their quest to understand P vs. NP: 1 Reduction Methods: • Cook-Levin Theorem: Introduced the concept of NP-completeness, showing that SAT (Boolean satisfiability) is NP-complete. • Karp's 21 NP-Complete Problems: Established many other problems as NP-complete through reductions. 2 Oracle Techniques: • Relativization: Considered whether P vs. NP holds relative to certain oracles, leading to limited insights. 3 Circuit Complexity: • Researchers have examined the complexity of boolean circuits and connections to P vs. NP. 4 Proof Complexity: • Investigating how hard it is to prove statements about P vs. NP within certain proof systems. 5 Algebraic Methods: • Using algebraic techniques and representations to study complexity classes. 6 Geometric Complexity Theory: • A recent approach combining algebraic geometry and representation theory.
**3**	Thank you for providing insights into the diverse approaches researchers have taken. In light of these strategies, my next question is more specific. Have there been any significant breakthroughs or advancements in recent years that brought us closer to resolving the P vs. NP problem, or are there notable challenges that still impede progress in this area?	As of my last knowledge update in September 2021, there were no significant breakthroughs leading to the resolution of the P vs. NP problem. Researchers continued to work on the problem, exploring various avenues and techniques, but the question remained open. Challenges that impede progress include the depth and complexity of the problem, the possibility that P vs. NP might be independent of standard axioms of set theory, and the need for new insights or techniques that have not yet been discovered. For the most recent developments, I would recommend checking the latest research papers and resources in the field of computational complexity theory. Progress in solving the P vs. NP problem is an ongoing and dynamic area of research.
**4**	Acknowledging the ongoing nature of the research, let's focus on the theoretical foundations. Given the challenges and the potential independence of P vs. NP from standard set theory axioms, are there any emerging or alternative mathematical frameworks that researchers are considering to reframe the problem or provide new perspectives?	Researchers have explored various mathematical frameworks and perspectives in their quest to address the P vs. NP problem. While I can't provide specific details on the latest developments, some of the frameworks and approaches that have been considered include: **Fine-Grained Complexity Theory:** Focusing on the exact running times of algorithms, which can provide insights into the nature of NP-hard problems. **Average-Case Complexity**: Studying the complexity of problems when input data is drawn from specific distributions, which can reveal nuances in the P vs. NP problem. **Algebraic Geometry and Geometric Complexity Theory**: These approaches use algebraic and geometric methods to explore complexity classes and their relationships. **Proof Complexity**: Investigating the complexity of proving statements about P vs. NP within various proof systems, which can shed light on the problem's nature. **Circuit Complexity and Proof Complexity Connections:** Examining the connections between circuit complexity and proof complexity to gain new insights. Researchers continue to explore these and other mathematical frameworks to gain fresh perspectives and potential breakthroughs regarding the P vs. NP problem. Stay updated with the latest research to learn about any emerging approaches or alternative frameworks.
**5**	Given the multifaceted nature of these approaches, it's evident that researchers are leveraging diverse mathematical tools to tackle the P vs. NP problem. In our final iteration, let's shift focus a bit. Considering the potential impact of a resolution to the P vs. NP problem, what practical applications and fields would be most profoundly affected if we were to discover that P equals NP or if they are proven to be distinct?	The resolution of the P vs. NP problem would have profound implications for various practical applications and fields: **Cryptography**:If P=NP, many cryptographic systems that rely on the difficulty of NP-hard problems (e.g., RSA) would become vulnerable to efficient attacks, posing significant security risks. If P ≠ NP, cryptography would be more secure, and new encryption methods may be developed. **Optimization**: Efficient algorithms for NP-complete problems could revolutionize optimization in numerous industries, including logistics, and manufacturing. **AI and Machine Learning:** A resolution could impact the development of AI algorithms, as many AI problems are NP-hard. If P=NP, it might lead to faster training and optimization of AI models. **Operations Research:** Decision-making and resource allocation in various fields, such as transportation and supply chain management, heavily rely on solving NP-hard problems. **Healthcare:** Drug discovery, protein folding, and other healthcare-related problems could benefit from efficient solutions if P=NP. **Data Analysis:** Many data analysis tasks involve NP-hard problems. A positive resolution could speed up data processing and analysis. **Scientific Research:**Problems in physics, biology, and other sciences that involve complex simulations or optimizations may become more tractable. In summary, a resolution of P vs. NP would have a wide-reaching impact, potentially revolutionizing fields that rely on solving complex problems, while also necessitating a reevaluation of security measures in cryptography.
**Conclusion**	Through our collaborative exploration, we've gained insights into the P vs. NP problem and its significance in computer science and mathematics. Key takeaways include: Problem Characteristics: P represents problems solvable in polynomial time. NP represents problems whose solutions can be verified in polynomial time. The unresolved question is whether P equals NP or P is distinct from NP. **Implications:** If P=NP, efficient algorithms exist for many challenging problems, impacting cryptography and optimization. If P ≠ NP, it suggests inherent computational limitations without proving specific problems are hard. **Approaches:** Reduction methods, circuit complexity, algebraic methods, and geometric complexity theory are among the diverse approaches researchers have explored. Ongoing challenges include the depth and complexity of the problem, potential independence from standard set theory axioms, and the need for new insights. **Recent Advancements:** As of my last update in September 2021, there were no significant breakthroughs leading to the resolution of P vs. NP. Researchers continue to explore various mathematical frameworks and perspectives, including fine-grained complexity theory, average-case complexity, algebraic geometry, and proof complexity. **Potential Impact:** Cryptography, optimization, AI, machine learning, operations research, healthcare, data analysis, and scientific research are among the fields that could be profoundly affected by a resolution to P vs. NP. While our exploration provides a snapshot of the current state of knowledge, it's essential to stay updated with the latest research for any breakthroughs or developments in this dynamic area of study.	

### 3.2 Scenario 2: automated

We have also automated such a communication mechanism using the APIs in Python, through which the two separate trained AIs communicate with each other using API calls and also, they can share responses to help each other in automating this process. Using API, limits the response with respect to the number of tokens. The respective implementation of this scenario is provided on GitHub^1^ for regeneration.

The initial prompt to initiate the conversation is:

“Try to find the Solution to the problem of P vs NP. Ask Questions to the Mathematics Expert?”

Table 2 lists the interaction between PG & PR for initially set ten iterations. We observe that even with the automated scenarios of generation of queries from PG and responses from PR, we are able to conclude at the end the certain solution to the problem provided at the initial prompt. Also, the size of responses is small, and the conclusion is clear and precise. Interestingly, the iterations and dialogues about the problem stops at seven, suggesting that if the PG and PR could reach to a conclusion, no further iterations of PG prompts are required.

**Table 2:** Interaction for scenario 2 between PG and PR.

Iterations	Prompt-Generator	Prompt-Responder
**1**	Could you explain the difference between problems that can be solved by P and those that are NP problems?	P problems refer to problems which are solvable in polynomial time, i.e. their computational complexity can be expressed using a polynomial function. Examples include problems such as the classic sorting algorithms, graph traversal, and balanced tree search. NP problems refer to problems which are solvable only in non-deterministic polynomial time, i.e. the best algorithms available are far slower than the polynomial-time algorithms used on P problems. Examples include travelling salesman problem, knapsack problem, and integer factorization.
**2**	What is the P vs NP problem?	The P vs NP problem is the open problem in computer science of determining whether or not a polynomial time algorithm exists that can solve any given NP problem. This is one of the most important unsolved problems in mathematics and computer science, with a potential breakthrough having implications for a wide range of application areas. If polynomial time algorithms can't be found, then it is possible that no practical solution exists for some NP problems, meaning the time required to solve them grows exponentially with the size of the problem.
**3**	Are there any techniques that can help solve the P vs NP problem?	There are numerous techniques being used by researchers to try and solve the P vs NP problem, such as approximation algorithms, heuristics, and exponential-time algorithms. Many researchers are also looking at the possibility of using quantum computing to tackle the problem as many of the most difficult problems can be solved more efficiently on a quantum computer. Additionally, researchers are also developing methods for creating efficient algorithms for particular problems and developing ways of analyzing the inherent complexity of a given problem.
**4**	What potential implications does the resolution of the P vs NP problem have?	The resolution of the P vs NP problem would have major implications on the fields of computer science and mathematics, as well as a range of application areas. A polynomial time algorithm for an NP problem could unlock the most difficult and intractable problems, allowing us to solve problems faster or to find an approximate solution more quickly. In addition, it could revolutionize computing by allowing us to access solutions to complex problems more quickly and efficiently.
**5**	Is there anything else I should know about the P vs NP problem?	Yes, the P vs NP problem is also closely related to the “P equals NP” problem, which involves determining if P is actually equal to NP. If P is indeed equal to NP, then all NP problems would be solvable in polynomial time, meaning that any problem that could be checked in polynomial time could also be solved in polynomial time. This would make many previously intractable problems solvable with more efficient algorithms, leading to implications for various fields of research and development.
**6**	What promising progress has been made on the P vs NP problem?	Over the years, researchers have made several important discoveries which could help provide a resolution to the P vs NP problem. In 2010, researchers found a proof which could potentially be used to show that certain classes of NP-complete problems could be solved in polynomial time. In 2016, there was a proof that certain NP-hard problems could also be solved in polynomial time. Unfortunately, no general polynomial time solution for all NP problems has been found yet, but these discoveries give us hope that a solution may one day be found.
**7**	Thank you for answering my questions. Is there any particular advice you have for approaching the P vs NP problem?	My advice for approaching the P vs NP problem is to focus on understanding the inherent complexity of the problem. It is important to analyze the problem closely, paying close attention to the underlying structure of the problem and the algorithms used to solve it. Furthermore, it is essential to stay up to date with the latest research and have an open mind to any new ideas that may arise, as this could key to finding a breakthrough resolution.
**8**	You're very welcome. Good luck with your research!	You're welcome.
**9**	Thank you!	
**10**		
**Conclusion**	The P vs NP problem is perhaps one of the most important open problems in mathematics and computer science, with massive implications for a wide range of application areas. P problems can be solved quickly using algorithms that run in polynomial time, while NP problems require non-deterministic algorithms that run in exponential time. Potential progress has been made in recent years with the discovery of certain proofs which suggest certain classes of NP-complete problems could be solved in polynomial time, while certain NP-hard problems could also be solved in polynomial time. To make progress with resolving the P vs NP problem, researchers should focus on understanding the inherent complexity of the problem and develop better algorithms and data structures that can help	

### 3.3 Scenario 3

In this scenario, we included the role of CO in the collaborative framework between PG and PR. The dialogue flow is as follows: **PG – CO – PR – CO – PG.** The following conversation is again simulated on ChatGPT 3.5.

#### Initiation of dialogue

- Present the initial prompt by the human expert to the Prompt-Generator (PG).

**Human Expert Prompt:** “Initiate a dialogue to discuss the potential environmental impact of a new space exploration mission."

**PG Meta-Prompt:** “Frame a question to elicit information on the environmental impact of the space exploration mission."

**PG Objective:** “Understand and communicate the potential environmental consequences."

**PG Context:** “No prior information on the specific mission."

**Generated Prompt by PG:** “Ask about the expected environmental impact of the upcoming space exploration mission.

#### Response from prompt-responder (PR)

- Describe how the PR, acting as an expert, responds to the prompt generated by the PG.
- Highlight the PR's ability to interpret the prompt, consider the context, and provide relevant information.

**PR Meta-Prompt:** “Provide a detailed and balanced response to the question about the environmental impact."

**PR Prompt:** “Respond to the inquiry about the expected environmental consequences of the upcoming space exploration mission."

**PR Context:** “Leverages knowledge from prior space missions and environmental impact assessments."

**Response by PR:** “The environmental impact of the mission is expected to be minimal due to the use of advanced propulsion systems and adherence to strict environmental protocols."

#### Compliance-Officer (CO) role

- Illustrate the role of the CO in ensuring ethical and legal compliance.
- Show how the CO refines prompts and responses to align with ethical guidelines and legal considerations.

**CO Meta-Prompt**: “Review and refine prompts and responses to ensure ethical and legal compliance."

**CO Prompt:** “Refine the generated prompt to ensure it adheres to ethical guidelines and legal requirements."

**CO Context (Legal_Context):** “Includes considerations such as data privacy and compliance with space exploration regulations."

**Refined Prompt by CO**: “Ask about the anticipated environmental impact while respecting data privacy regulations."

#### Test run on ChatGPT instances

##### Iteration 1

PG: “Initiate a dialogue to discuss the potential environmental impact of a new space exploration mission."

CO: “Refine the generated prompt to ensure it adheres to ethical guidelines and legal requirements."

PR: “The potential environmental impact of the new space exploration mission depends on factors such as propulsion systems, waste management, and adherence to environmental protocols."

##### Iteration 2

PG: “Explore the specific measures taken to minimize space debris in the proposed space mission."

CO: “Refine the generated prompt to seek information on measures to minimize space debris, ensuring compliance with space exploration regulations."

PR: “To minimize space debris, the mission includes advanced debris removal systems and adherence to international space debris mitigation guidelines."

##### Iteration 3

PG: “Inquire about the use of AI technologies in the proposed space mission and any ethical considerations related to AI."

CO: “Refine the generated prompt to ensure it addresses ethical considerations related to the use of AI in the proposed space mission, complying with ethical guidelines."

PR: “The use of AI in the mission raises ethical considerations, including transparency in AI decision-making and potential biases that need to be carefully addressed."

##### Iteration 4

PG: “Ask about the potential impact of the mission on local ecosystems and wildlife."

CO: “Refine the generated prompt to ensure it considers the potential impact on local ecosystems and wildlife, complying with environmental regulations."

PR: “The mission undergoes rigorous environmental impact assessments to mitigate any potential negative effects on local ecosystems and wildlife."

##### Iteration 5

PG: “Discuss the role of international collaboration in ensuring responsible space exploration practices."

CO: “Refine the generated prompt to specifically address the importance of international collaboration in ensuring responsible space exploration, aligning with global ethical standards."

PR: “International collaboration is vital to fostering responsible space exploration, sharing best practices, and ensuring that missions adhere to ethical and legal standards globally."

##### Iteration 6

PG: “Inquire about the steps taken to protect sensitive data related to the space mission."

CO: “Refine the generated prompt to ensure it includes considerations for protecting sensitive data, complying with data protection regulations."

PR: “Sensitive data related to the space mission is protected through robust encryption, access controls, and compliance with data protection regulations."

##### Final conclusion

In conclusion, the comprehensive approach taken by the new space exploration mission reflects a commitment to ethical, legal, and environmental considerations. By addressing potential risks and incorporating best practices, the mission aims not only to advance scientific exploration but also to set a precedent for responsible and sustainable space endeavours. This dialogue, guided by ethical refinement from our Compliance-Officer, ensures that the mission aligns with the highest standards of conduct in the realms of space exploration, technology, and environmental stewardship.

From this scenario, we infer that the integration of CO between PG and PR demonstrates the value of assimilating ethical and legal compliance into collaborative frameworks between AI-AI. The PR suitably interprets prompts and provide informed responses within the given context. The dialogue further shows a commitment to responsible practices, which adhere to the ethical, legal, and environmental considerations in space exploration.

## 4 Discussion, conclusion, and future works

This responsible AI-AI conversation paradigm can serve as transformative technology that has the potential to reshape a wide range of industries, economies, and societies. The proposed framework provides a defined structure where we have back-and-forth interaction between generating prompts, responding to prompts, and enforcing compliance to attain a solution to the bigger problem. It can be assumed as an interesting analogy of a “game” with a rule-enforcing arbiter. The proposed conceptualization could offer insights into designing and refining the interactions within the AI-AI dialogue system. Here are a few inferences and implications of the proposed architecture:

• The “Prompt-Generator vs. Prompt-Responder” pair guarantees “Autonomy” of decision-making processes.
• the framework can help in organizing and understanding the dialogue flow between two autonomous AI systems.
• The inclusion of a CO will ensure compliance with rules and regulations. This has significance especially in the applications with legal implications and ensuring the responsible use of AI.
• Collaborative work of the whole triplet of those provide decision-making scenarios of “Responsible Autonomy".

**Fig. 4:**
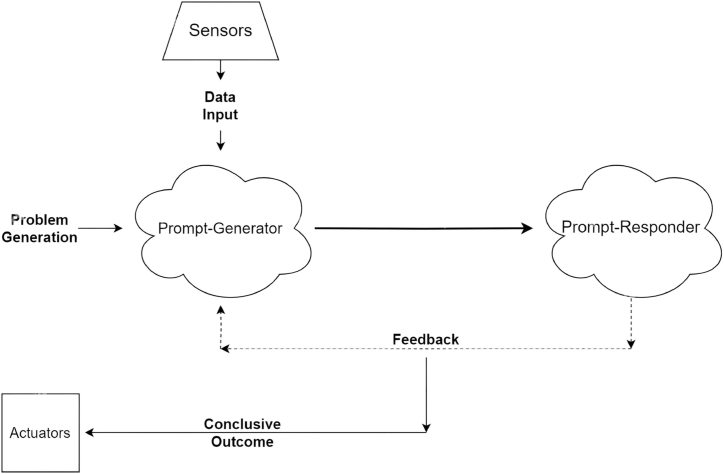
Real-world implication of the AI as a user of itself.

This AI-AI collaboration tries to address the major objective of problem-solving for complex scenarios. However, it's essential to address the visible challenges such as consistency and rationality in the conversation so that both the AI models understand and interpret the prompts correctly, thus, also addressing the biases that might exist in their responses to the prompts. Several other challenges and considerations are as follows:

• It's essential to carefully consider the high-level overall objective, stage-specific objectives, and the context of ongoing and previous interactions for context-awareness of the actions of PG and PR and thus establishing the coherence in this AI-AI collaborative ecosystem.
• In addition to that, it will be critical as well to monitor the conversation flow so that this AI-AI system converges to a meaningful and accurate outcome.
• Another important aspect for the successful deployment of this approach is better prompt engineering, which involves precise and clear prompts as an input to the AI-model (prompt-generator in our case) to direct their behaviour.
• A “well-engineered” prompt can generate refined problem statements and thus relevant responses from the prompt-responder [27,28]. As has been seen in the medical research domain, prompt engineering has the potential to influence how efficiently and effectively healthcare can be delivered to mankind. The healthcare providers can sensibly use prompts to fine-tune AI models for specific tasks, enable access to accurate information, and analyze big data for predicting trends in medical diagnosis [27].
• The team at OpenAI, responsible for chatGPT, is working on a highly autonomous self-sufficient system called the superintelligence [29] with the goal of surpassing human intelligence and addressing global challenges. However, the most important challenge is to align this superintelligence-enabled AI system with the human intent. Also, there is an understandable risk of severe consequences for humanity, if not developed properly keeping in mind the alignment with human values, social and economic disturbances, ability to tackle unforeseen circumstances, etc.
• Fig. 4 envisions a real-world scenario for the proposed architecture, where the AI system becomes its own user and addresses a real-world problem. By integrating sensors, the AI architecture gathers real-time data from the environment. PG, then, initiates the prompts that encapsulate the specific challenges or scenarios presented by the sensory data or some problem at hand. Accordingly, the PR responds by forming a dynamic dialogue which is solely aimed at problem-solving. This interactive process synergies with the above-discussed concept of “Responsible Autonomy”. By connecting actuators, the AI system can execute the proposed solutions in the real world and thus the feasibility of the proposed architecture is enhanced. This particular scenario addresses the adaptability of the AI-AI collaborative ecosystem and demonstrates its potential to address real-world challenges by utilizing sensor data, exchanging meaningful dialogues, and finally translating the conclusion into effective actions through actuators. The self-contained decision-making capabilities of the proposed architecture present a futuristic vision for advancing AI applications in the real world, which can address problems in wide range of domains such as indoor positioning [30], robotics [31], synthetic aperture radar [32], scheduling [33], etc.

• The proposed framework acknowledges the robust scalability and integration capabilities of ChatGPT 3.5. Mechkaroska et al. [34] highlights ChatGPT's architectural scalability which is crucial for efficiently handling rising computational demands without compromising performance. ChatGPT's underlying architecture and distributed computing abilities enable seamless integration with diverse systems and technologies, including biomedical research and healthcare applications [35]. Ray [36] provided a comprehensive review outlining potential challenges, biases, and limitations associated with ChatGPT. However, these limitations are subject to ongoing improvement and refinement as the field progresses.

The future prospect of the proposed architecture suggests that whenever there will be a more effective AI (such as [29]) surpassing human capabilities, it will comprehend a PG, PR, and CO which will also exhibit capabilities outperforming their human complements. Eventually, it will result in the “Responsible Autonomy of AI,” exceeding the responsible autonomy exhibited by humans. The future work shall investigate strategies for ensuring data privacy and compliance with regulations such as General Data Protection Regulation (GDPR), which can be handled by the AI based CO. It's also essential to recognize the importance of addressing the need for continuous ethical and legal progression in the rapidly evolving landscape of Generative AI domain. As AI systems become more autonomous and capable [29], it will be necessary to align with ethical guidelines and regulatory frameworks. Future work may also address strategies for staying well-informed with the ethical and legal developments and integrating them into the design and deployment of the proposed framework. Furthermore, the issues with more complex problem domains and additional approaches to enhance the scalability is also a potential area to explore.

## Data availability statement

Data included in study/supplementary material/referenced in study.

## CRediT authorship contribution statement

**Amit K. Shukla:** Writing – original draft, Visualization, Methodology, Investigation, Formal analysis, Conceptualization. **Vagan Terziyan:** Writing – review & editing, Supervision, Methodology, Investigation, Conceptualization. **Timo Tiihonen:** Writing – review & editing, Supervision, Methodology, Conceptualization.

## Declaration of competing interest

The authors declare the following financial interests/personal relationships which may be considered as potential competing interests: First Author is the Associate Editor of the Journal. Other authors declare that they have no known competing financial interests or personal relationships that could have appeared to influence the work reported in this paper.

## Footnotes

1 https://github.com/amitkshuklaa/AIvsAI.
